# Spectrum of Disease Severity in Patients With X-Linked Retinitis Pigmentosa Due to *RPGR* Mutations

**DOI:** 10.1167/iovs.61.14.36

**Published:** 2020-12-29

**Authors:** Valentina Di Iorio, Marianthi Karali, Paolo Melillo, Francesco Testa, Raffaella Brunetti-Pierri, Francesco Musacchia, Christel Condroyer, John Neidhardt, Isabelle Audo, Christina Zeitz, Sandro Banfi, Francesca Simonelli

**Affiliations:** 1 Eye Clinic, Multidisciplinary Department of Medical, Surgical and Dental Sciences, Università degli Studi della Campania “Luigi Vanvitelli,” Naples, Italy; 2 Telethon Institute of Genetics and Medicine, Pozzuoli, Italy; 3 Medical Genetics, Department of Precision Medicine, Università degli Studi della Campania “Luigi Vanvitelli,” Naples, Italy; 4 Sorbonne Université, INSERM, CNRS, Institut de la Vision, Paris, France; 5 Human Genetics, Faculty of Medicine and Health Sciences, University of Oldenburg, Oldenburg, Germany; 6 Research Center Neurosensory Science, University Oldenburg, Oldenburg, Germany; 7 CHNO des Quinze-Vingts, DHU Sight Restore, INSERM-DGOS CIC, France; 8 Institute of Ophthalmology, University College of London, London, United Kingdom

**Keywords:** *RPGR*, X-linked retinitis pigmentosa, sine pigmento retinitis pigmentosa, myopia, ORF15

## Abstract

**Purpose:**

The purpose of this study was to perform a detailed longitudinal phenotyping of X-linked retinitis pigmentosa (RP) caused by mutations in the *RPGR* gene during a long follow-up period.

**Methods:**

An Italian cohort of 48 male patients (from 31 unrelated families) with *RPGR*-associated RP was clinically assessed at a single center (mean follow-up = 6.5 years), including measurements of best-corrected visual acuity (BCVA), Goldmann visual field (GVF), optical coherence tomography (OCT), fundus autofluorescence (FAF), microperimetry, and full-field electroretinography (ERG).

**Results:**

Patients (29.6 ± 15.2 years) showed a mean BCVA of 0.6 ± 0.7 logMAR, mostly with myopic refraction (79.2%). Thirty patients (62.5%) presented a typical RP fundus, while the remaining sine pigmento RP. Over the follow-up, BCVA significantly declined at a mean rate of 0.025 logMAR/year. Typical RP and high myopia were associated with a significantly faster decline of BCVA. Blindness was driven primarily by GVF loss. ERG responses with a rod-cone pattern of dysfunction were detectable in patients (50%) that were significantly younger and more frequently presented sine pigmento RP. Thirteen patients (27.1%) had macular abnormalities without cystoid macular edema. Patients (50%) with a perimacular hyper-FAF ring were significantly younger, had a higher BCVA and a better-preserved ellipsoid zone band than those with markedly decreased FAF. Patients harboring pathogenic variants in exons 1 to 14 showed a milder phenotype compared to those with *ORF15* mutations.

**Conclusions:**

Our monocentric, longitudinal retrospective study revealed a spectrum disease progression in male patients with *RPGR*-associated RP. Slow disease progression correlated with sine pigmento RP, absence of high myopia, and mutations in *RPGR* exons 1 to 14.

Retinitis pigmentosa (RP) is the most common form of inherited retinal dystrophy with a prevalence of 1:3500 to 1:4000.^1^ The main cellular hallmark of RP is a progressive degeneration of photoreceptors that first leads to night blindness and loss of peripheral visual field, and subsequently may cause legal blindness.^1^ RP is more frequently observed as an isolated form (also called simplex RP), without extra-ocular involvement.^2^ It is characterized by high genetic heterogeneity, with over 130 genes reported to cause either isolated (approximately 90 genes) or syndromic forms (approximately 40 genes; http://www.sph.uth.tmc.edu/RetNet; accessed on April 2020). The majority of RP genes follow an autosomal recessive inheritance (AR). Nevertheless, autosomal dominant (AD) and X-linked (XL) patterns of inheritance (XLRP) are observed in a significant number of cases.^2^

Approximately 15% of RP cases are inherited as XL traits and are usually associated with both an earlier onset and a more severe phenotype.^3^^–^^5^ Male patients with XLRP usually present night blindness, myopia (often since childhood), progressive loss of peripheral visual fields, and subsequent loss of central vision in relation with severe rod and cone dysfunction.^6^ Disease progression in male patients with XLRP is one of the most rapid among all RP, reaching legal blindness by the third to the fourth decade.^5^^,^^7^ Other clinical hallmarks of XLRP include bone spicule pigmentary changes, attenuated retinal blood vessels, optic disc pallor, visual field loss, and diminished or non-recordable electroretinogram (ERG) responses.^8^ Female carriers show a spectrum of clinical manifestations that range from asymptomatic to severe, which can in some instances lead to the misdiagnosis of an AD mode of inheritance based on the evaluation of the family pedigree.^9^^–^^12^ The main genes associated with XLRP are the Retinitis Pigmentosa GTPase Regulator gene (*RPGR*; OMIM # 312610), accounting for up to 80% of XLRP cases,^5^^,^^13^^,^^14^ and the Retinitis Pigmentosa 2 (*RP2;* OMIM # 312600) responsible for approximately 5% to 15% of cases.^15^ Additional genes account only for a limited fraction of XLRP cases. For example, a severe form of XLRP (RP23) in a single family was associated with a deep intronic variant in the OFD1 Centriole and centriolar satellite protein (*OFD1*; OMIM # 300170), which is most commonly responsible for the X-linked dominant Oral-facial-digital syndrome type 1.^16^ Moreover, XLRP combined with variable degrees of neurological symptoms and hearing loss was reported in women heterozygous for a novel loss-of-function mutation in the phosphoribosyl pyrophosphate synthetase 1 gene (*PRPS1*; OMIM # 311850).^17^

*RPGR* was identified by positional cloning in the *RP3* locus on chromosome Xp11.4 (OMIM # 300029).^18^^,^^19^ The *RPGR* gene has 19 exons and undergoes extensive alternative splicing that generates at least ten different isoforms.^14^^,^^20^^–^^23^ The two main *RPGR* isoforms are the constitutively expressed *RPGR*,^1^^–^^19^ composed of 19 exons and encoding an 815-aa protein, and the retina-specific *RPGR^ORF15^* isoform, composed of 15 exons and encoding a 1152-aa protein.^18^ Both isoforms share exons 1 to 14, encoding a regulator of chromosome condensation 1 (RCC1)-like domain essential to interact with binding partners,^24^ but diverge at their carboxyl-terminal ends. In particular, the terminal exon (open reading frame 15; *ORF15*) of the *RPGR^ORF15^* isoform contains a repetitive, purine-rich sequence coding for a low complexity stretch of 567-aa with a high glutamic acid and glycine (Glu/Gly) content.^23^ Until now, more than 350 sequence variants have been described in *RPGR*^25^ and almost 60% of them are found in *ORF15*.^26^ The high mutation frequency in *ORF15* (mainly small deletions or duplications) is most likely due to the nucleotide composition and repetitive nature of its sequence, which might induce errors during DNA replication.^27^

To date, genotype-phenotype correlations have been investigated in several *RPGR* patient cohorts, yet a consensus remains to be reached. Studies suggested a more severe RP phenotype in patients with mutations in exons 1 to 14 compared to patients with variants in *ORF15*.^7^^,^^8^^,^^28^^–^^30^ However, other reports described a milder clinical presentation in patients carrying variants in exons 1 to 14 compared to those with *ORF15* mutations.^31^^,^^32^ A common ground was found instead regarding *RPGR* variants located toward the 3′ end of *ORF15*, which were more frequently associated with milder XL Cone Rod Dystrophy (CRD) / XL Cone Dystrophy (CD) phenotypes.^25^^,^^30^^,^^33^^–^^35^ Finally, some *RPGR* variants in exons 1 to 14 were reported in patients with RP presenting progressive hearing loss, sinusitis, and chronic recurrent respiratory tract infections,^36^^–^^38^ in line with the more widespread expression of the *RPGR*^1^^–^^19^ isoform and its essential ciliary function in other organs apart from retina.^39^^,^^40^

In this study, we report the clinical and genetic findings in a cohort of 48 male patients of Italian origin with XLRP caused by variants in *RPGR.* We describe the spectrum of retinal phenotypes encountered in this cohort and the longitudinal progression of *RPGR*-associated RP. We also sought to identify clinical parameters that could reliably predict disease progression. In that respect, our observations gain strength by the fact that all subjects were diagnosed and followed up at a single center. Ultimately, these results can be useful for selecting and prioritizing patients in clinical trials, as well as for establishing effective read-outs to assess treatment outcomes.

## Methods

### Ethics Statement

All procedures adhered to the tenets of the Declaration of Helsinki and were approved by the Ethics Board of the Università degli Studi della Campania “Luigi Vanvitelli” (for adult protocol no. 8189/2015, 09.04.2015; for pediatric subjects’ protocol no. 500/2017, 12.09.2017). The cited protocols aimed to investigate genotype-phenotype correlations in inherited retinal dystrophies. Peripheral blood samples were collected upon written informed consent of the subjects for sample collection and genetic analysis. For minors, informed consent was obtained by the parents or legal guardians.

### Patients’ Inclusion Criteria

In this study, we analyzed subjects who were recruited in the above-cited protocols and satisfied the following inclusion criteria: only male patients with a clinical diagnosis of RP and disease-causing variants in the *RPGR* gene. Therefore, we excluded female subjects (i.e. obligate female carriers, which usually show no or limited signs of the disease) and patients with *RPGR*-associated CD or CRD. Patients belonging to the same family were included in the study and assigned the same family identification number.

The clinical diagnosis of RP was formulated according to the criteria described by Hamel.^1^ Specifically, the diagnosis requires a dramatic reduction in a- and b-wave amplitudes at the ERG (with a rod-cone pattern of photoreceptor dysfunction at the early stages of the disease) associated with one or more of the following alterations: night blindness and/or photophobia even with preserved visual acuity in the early and mid-stages of the disease; visual field changes such as irregular loss of peripheral visual field evolving to ring scotoma, and eventually to tunnel vision; fundus abnormalities, such as pigmentary deposits resembling bone spicules, initially in the mid peripheral retina (hereafter referred to as typical RP fundus appearance), an attenuation of the retinal vessels, as well as waxy pallor of the optic disc accompanied by various degrees of retinal atrophy at the fundus examination. Based on the fundus appearance, RP is also defined as sine pigmento, sector, and pericentral on the basis of presence and localization of bone spicule pigmentary deposits.^1^^,^^41^

Disease onset was defined as the self-reported age of the first symptoms (i.e. night vision problems and/or visual abnormalities). The disease length was calculated over time between the self-reported onset of symptoms and the date of the examination. In case of incidental diagnosis, the age at diagnosis was considered as the disease onset.

### Ophthalmological Examination

The medical records of all the included subjects seen at the Referral Center for Inherited Retinal Dystrophies of the Eye Clinic, University of Campania “Luigi Vanvitelli” were reviewed to extract the findings of the following ophthalmological examinations: best-corrected visual acuity (BCVA) measurements with the Snellen visual chart, slit lamp anterior segment examination, measurements of intraocular pressure, fundus examination, Goldmann visual field (GVF) examination, optical coherence tomography (OCT), Fundus Autofluorescence (FAF), Microperimetry (MP1), and standard full-field ERGs.

Spherical equivalent, defined as the sum of the sphere and one half the cylinder power and averaged between the two eyes, was used to calculate refractive error in diopters (D). Refractive errors were classified into high myopia (≤ −6 D), moderate myopia (> −6 D to ≤ −3 D), low myopia (< −3 D to ≤ −0.75 D), emmetropia (> −0.75 D to < 0.75 D), low hyperopia (≥ 0.75 D to < 3 D), medium hyperopia (≥ 3 D to < 6 D), and high hyperopia (≥ 6 D), using the criteria defined by the CREAM consortium (CREAM consortium meeting, 2012, Sardinia, Italy).

GVF was measured by moving the III4e and V4e stimulus target on a calibrated standard Goldmann perimeter by the same experienced ophthalmic technician. Legal blindness based on GVF was determined following the indications by the International Classification of Diseases (ICD-10 Version 2016; International Statistical Classification of Diseases and Related Health Problems 10th Revision [ICD-10]-World Health Organization [WHO] Version 2016; i.e. central III4e GVF area in the better-seeing eye no greater than 314 degrees,^2^ corresponding to an equivalent radius of 10 degrees).

OCT was performed by experienced operators either using the Heidelberg Spectralis OCTPlus with BluePeak (Heidelberg Engineering, Heidelberg, Germany) or the Cirrus HD-OCT (Carl Zeiss, Dublin, CA, USA). The macular abnormalities (MAs) revealed by OCT scans were classified for cystoid macular edema (CME), vitreo-macular traction syndrome (VMT), epiretinal membrane (ERM), full-thickness macular hole (FTMH), lamellar macular hole (LMH) and tractional macular edema (TME), as previously described.^42^ The mean macular thickness (MMT) and the length of the ellipsoid zone band (EZ)^43^^,^^44^ (previously known as the IS/OS line) were measured using SD-OCT performed on the Heidelberg Spectralis OCTPlus with the BluePeak imaging platform. The measurements were performed manually by two operators and revised by a third one in case of discrepancies. FAF imaging of the 30 degrees field was acquired with the Heidelberg Spectralis OCTPlus with BluePeak (Heidelberg Engineering, Heidelberg, Germany). Following previous studies,^34^^,^^45^ the FAFs were classified on the basis of the presence of a hyperautofluorescent ring or an abnormal central hypoautofluorescence.

MP1 was performed by an automatic fundus-related perimeter (MP1 Microperimeter, Nidek Technologies, Padova, Italy). The following parameters were used: a fixation target of 2 degrees in diameter consisting of a red ring; a white, monochromatic background with a luminance of 1.27 cd/m²; a Goldmann III-size stimulus with a projection time of 200 ms; and a predefined automatic test pattern (Humphrey 10-2) covering 10 degrees centered onto the gravitational center of the fixation points with 68 stimuli.

Full-field ERG was recorded using corneal contact lens electrodes with a Ganzfeld stimulator (Roland Consult, Brandenburg, Germany) according to the standards of the International Society for Clinical Electrophysiology of Vision.^46^

### Next Generation Sequencing and Variant Interpretation

Genomic DNA was extracted from peripheral blood using the DNeasy Blood & Tissue Kit (QIAGEN) according to the manufacturer's instructions. Panel-based sequencing (RETplex) was performed as previously described.^47^ Libraries for clinical exome and whole-exome sequencing were prepared using the ClearSeq Inherited Disease Panel (Agilent Technologies) and the SureSelect Human All Exon V7 (Agilent Technologies), respectively. Targeted regions were enriched using the SureSelectQXT Target Enrichment system (Agilent Technologies). Libraries were run on a NextSeq500 sequencing platform (Illumina Inc., San Diego, CA, USA). Sequencing data were analyzed using an already reported pipeline.^48^ Only variants with a minor allele frequency (MAF) < 0.05 in the Genome Aggregation Database (gnomAD; http://gnomad.broadinstitute.org) were considered. Variant interpretation and analysis was performed as previously described.^49^ The identified variants were validated by Sanger sequencing of the corresponding genomic fragments. For the amplification of the exon sequence, PCR was performed on 20 ng of genomic DNA using AmpliTaq Gold polymerase (Thermo Fisher Scientific) according to standard protocols. Amplicons were Sanger sequenced and sequences were aligned to the reference genome (hg19) using the UCSC Genome Browser (http://genome.ucsc.edu/). Mutation detection was performed using the CodonCode Aligner software. The novel variants identified in this study have been deposited to the Leiden Open Variation Database (LOVD version 3.0; https://www.lovd.nl/).

### *ORF15* Amplification and Sanger Sequencing

The *ORF15* sequence was analyzed similarly to previously described procedures.^50^ We amplified the *ORF15* genomic region (NM_001034853.1) by PCR on 200 ng genomic DNA using the HOT FIREPol DNA Polymerase (Solis BioDyne) and the oligonucleotide primers R1Ampl and R2Ampl (Supplementary Table S1). Solution S (Solis BioDyne) was added in the PCR mix at a final concentration of 3X. The following cycling parameters were used: an initial denaturation step (95°C, 15 minutes) followed by 45 cycles at 95°C for 45 seconds, 60°C for 1 minute and 72°C for 3 minutes and 30 seconds, with a final extension of 10 minutes at 72°C. After a PCR clean-up step (ExoSAP-IT), amplicons were Sanger sequenced using the BigDye Terminator version 1.1 Cycle Sequencing Kit (Thermo Fisher Scientific) and the following primers: R7bSeq, R8bSeq, R9Seq, R5Seq, R4Seq, and R11Seq (Supplementary Table S1). Annealing and extension temperatures are reported in Supplementary Table S1.

### Criteria for Genotype-Phenotype Correlation

To explore possible genotype-phenotype correlations, patients’ genotypes were stratified according to the position of the variants in the major *RPGR* isoform (i.e. variants in exons 1–14 versus variants in *ORF15*). Variants in exons 1 to 14 were further classified according to the mutation type (i.e. null variants, splice variants, missense variants). Regression models were applied as detailed below.

### Statistical Analysis

Continuous variables are reported as mean ± standard deviation (SD) and categorical variables are reported as counts (percentage).

The natural history of disease was analyzed using previously applied methods.^51^^,^^52^ In order to include all the patients (i.e. also those with no longitudinal data), we performed a cross-sectional analysis on the data of the last visit for each of the selected outcome measures (e.g. BCVA, MMT, and EZ band width). In particular, linear regression models, estimated by a generalized estimating equation (GEE), were performed with each outcome measure as the dependent variable and age as the independent variable in order to estimate a slope (mean rate of change per year of age) with its relative standard error (SE).

Moreover, regression models were fitted with each outcome measure as the dependent variable and high myopia, sine pigmento fundus, gene region harboring the mutation (exons 1–14 versus *ORF15*), mutation type (i.e. null variants in exons 1–14, splice variants in exons 1–14, or variants in *ORF15*) as independent variable, in order to investigate whether these factors influenced the selected outcome. We also controlled for age (added as an additional independent variable) when a variable was shown to be age-related. To this regard, the Student's *t*-test was used to evaluate if the mean age of the patients, self-reported age of onset, and disease length were different between the following subgroups: patients with high myopia versus those without high myopia; patients with typical RP versus patients with sine pigmento; and patients with variants in exons 1 to 14 versus those with variants in *ORF15*. Analysis of variance (ANOVA) with Bonferroni correction for multiple comparisons performed with *t*-tests was adopted to evaluate differences in the age between the patients classified according to lens status and according to the mutation type. Fisher exact tests and Pearson χ^2^ tests were adopted to explore differences for dichotomous and categorical variables, respectively. For dichotomous or categorical variables related to age, logistic regression models were fitted including age as an additional independent variable.

Moreover, GEE were fitted on longitudinal data, using baseline values as offset and follow-up length expressed in years as an independent variable, to estimate the annual change of the BCVA, MMT, and the EZ band width over the follow-up period. The analysis of BCVA was conducted separately in the best-seeing eyes and in the worst-seeing eye in order to assess the symmetry in disease progression. Moreover, asymmetry in BCVA between the two eyes was defined as a difference of 0.3 logMAR (15 Early Treatment Diabetic Retinopathy Study [ETDRS] letters), which is the threshold for clinical significance of BCVA changes.^53^ Finally, regression models, including the interaction between follow-up length and the following factors: high myopia, sine pigmento fundus, gene region affected by the mutation, and mutation type, were fitted in order to investigate whether these factors influenced the decline of BCVA. We controlled for age (added as an additional independent variable) when a factor has been shown to be age-related.

GEE were applied because this method could accommodate the inter-eye correlation (i.e. between the two eyes of the same subject at a given visit) and longitudinal correlation (i.e. between values of the same eye followed over time) by adopting an appropriate covariance structure, as previously described.^54^

For the statistical analysis, BCVA were converted to logMAR using the values of 2.7 for hand motion, 2.8 for light perception, and 2.9 for no light perception. All the other measures (e.g. MMT and EZ band width) were also log-transformed to estimate the exponential rate of progression. Intercepts were included in all the models, except those regarding BCVA, because we assume that BCVA should start with the value of 0 logMAR (corresponding to healthy status).

Finally, a Kaplan-Meier survival analysis was performed to show the time to low vision, blindness based on visual acuity, and blindness based on visual field. We adopted the definition proposed by the ICD-10 version 2016: BCVA worse than 20/67 in the better-seeing eye for low vision; BCVA worse than 20/400 in the better-seeing eye for blindness based on visual acuity; III4e GVF area in the better-seeing eye no greater than 314 degrees^2^ (i.e. corresponding to an equivalent radius of 10 degrees) for blindness based on visual field. The relative distributions were compared with the Log Rank (Mantel-Cox) test.

For the analyses involving only clinical parameters, all the patients were included assuming that the observations could be considered as independent given the known intrafamiliar variability. On the other hand, the analyses involving genotype were performed only on unrelated patients. In other words, only one patient from each family was included (i.e. the first diagnosed patient; assigned the lowest ID number among the family components). Related patients were excluded to reduce the risk of findings biased toward families with higher numbers of affected subjects.

The *P* values (*P*) lower than 0.05 were considered statistically significant. The reported *P* values relative to the *t*-tests incorporate the Bonferroni correction in case of multiple comparisons. These statistical analyses were performed using the IBM SPSS Statistics platform (version 21.0.0.0).

## Results

### Clinical Characterization of the *RPGR*-RP Cohort

A total of 48 patients (mean age = 29.6 ± 15.2 years) from 31 families with a clinical diagnosis of RP and harboring a disease-causing mutation in the *RPGR* gene were involved in this clinical study. Two patients with disease-causing mutations in the *RPGR* gene but with a clinical diagnosis of CD were not included in the above cohort as they did not meet the inclusion criteria.

**Figure 1.**
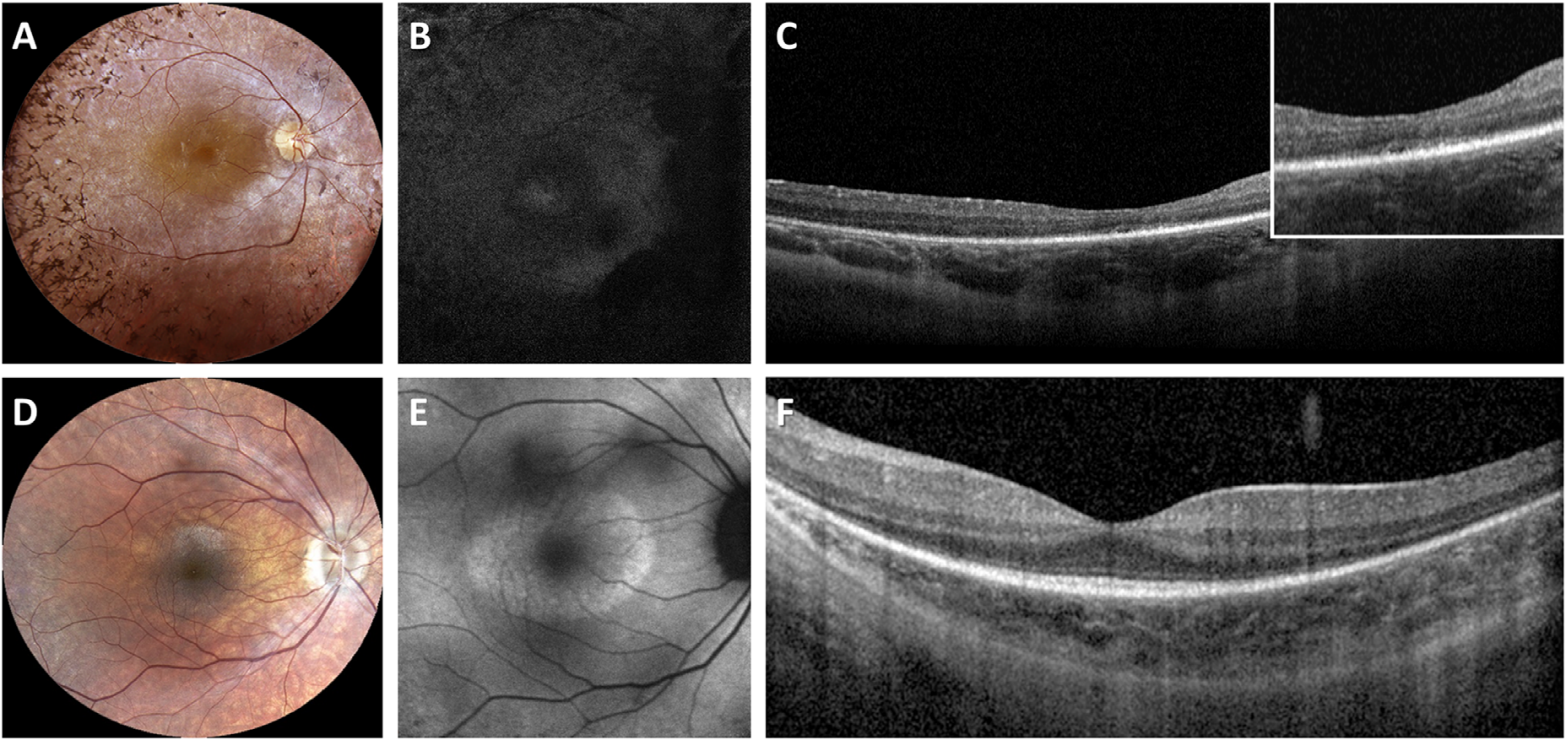
Multimodal imaging findings in two representative patients with *RPGR*-associated RP. (**A**) Fundus photograph of a 31-year-old patient with typical RP (ID no. 13; BCVA 0,3; NM_001034853: c.1245+3A>T) showing bone-spicule pigment deposits in the mid peripheral retina, optic disc pallor and RPE atrophy. (**B**) Fundus autofluorescence (FAF) image showing a widespread hypoautofluorescence. (**C**) Spectral domain OCT revealing RPE atrophy. The inset shows the disruption of the ellipsoid zone (EZ) band. (**D**) Fundus photograph of a 25-year-old patient with sine pigmento RP (ID no. 37; BCVA 0,7; NM_001034853: c.1059+1G>A) showing absence of bone-spicule-like pigment migration, optic disc pallor, and mild RPE atrophy. (**E**) FAF image showing a hyperautofluorescent ring around the central macula. (**F**) Spectral domain OCT showing central sparing of the EZ band that is attenuated toward the peripheral macula.

The main clinical findings of the cohort are summarized in Table 1. The age at onset of the first symptom was 6.9 ± 4.6 years (median = 5 years; range = 1–21 years; interquartile range = 2.5 years). Almost all patients (45; 93.8%) reported that night blindness was present at disease onset, whereas reduction of visual field (12; 25.0%) and decreased visual acuity (5; 10.4%) were less frequently observed at disease onset. A typical RP fundus was found in the majority of patients (30; 62.5%), whereas the sine pigmento RP form was detected in 37.5% of patients (*n* = 18; Fig. 1). Patients with sine pigmento RP were significantly younger compared to patients with a typical RP fundus (20.4 ± 6.9 years versus 35.1 ± 16.2 years; *P* = 0.001).

**Table 1.** Clinical Findings in the Patients With *RPGR-*associated RP

Parameters	Study Cohort (*n* = 48)	
Age, y	29.6 ± 15.2	
Self-reported age of onset, y	6.9 ± 4.6	
Disease length, y	22.7 ± 15.4	
Mean refractive error (D)	−4.0 ± 3.1	
High myopia	16 (33.3%)	
Typical RP	30 (62.5%)	
Vitreomacular alteration	13 (27.1%)	
Detectable photopic ERG	24 (50.0%)	
	Right Eye	Left Eye
BCVA, logMAR	0.63 ± 0.79	0.62 ± 0.74
EZ-band width, µm	1319.0 ± 685.9	1326.0 ± 571.9
MMT, µm	226.7 ± 43.1	224.6 ± 44.4
Photopic ERG (b-wave amplitude), µV	20.7 ± 14.0	24.0± 17.5
Photopic ERG (b-wave implicit time), ms	43.5 ± 2.8	43.1 ± 4.4
30 Hz Flicker ERG (trough-to-peak amplitude), µV	11.8 ± 9.1	11.7 ± 10.7
30 Hz Flicker ERG (implicit time), ms	46.6 ± 8.5	45.8± 8.0
MS, dB	2.6 ± 3.3	3.8 ± 4.4

The most recently measured mean spherical equivalent, averaged between eyes, was −4.0 ± 3.1 D (range = −12 D to +1.25 D). Most patients (39; 79.2%) were myopic. In particular, high, moderate, and low myopia were observed in 16 (33.3%), 12 (25.0%), and 11 (22.9%) patients, respectively. Eight patients (16.7%) were emmetropic and only one patient showed a low hyperopia (+1.25 D in both eyes). There was no statistically significant difference (*P* = 0.058) in the frequency of high myopia between typical RP and sine pigmento RP. Cataract or pseudophakia was found in 21 patients (43.7%) and we observed a significant association between cataract/pseudophakia and age (*P* < 0.001). Specifically, patients with clear lenses in both eyes were younger (20.1 ± 6.3 years) than those with cataract (34.1 ± 8.3; *P* < 0.001) or patients with pseudophakia (52.0 ± 15.3 years; *P* < 0.001).

In the study cohort, at the most recent visit, mean BCVA was 0.63 ± 0.79 logMAR in the right eyes and 0.62 ± 0.74 logMAR in the left eyes. The analysis of inter-eye asymmetry values showed a low asymmetry in the BCVA (median = 0.06 logMAR, equivalent to 3 ETDRS letters) with only eight patients (16.7%) recording higher values than the established threshold for clinically significant changes (i.e. 0.3 logMAR).

Longitudinal analysis of BCVA data collected in 41 patients over a mean follow-up period of 6.5 years (SD = 4.4; range = 1–18 years; median = 5 years; average number of visits = 5), reported in Table 2, showed a significant decline of 0.025 logMAR/year (SE = 0.012; *P* = 0.047) equivalent to about 1 ETDRS letter/year in the best-seeing eyes. A faster decline was observed in the worst-seeing eyes (i.e. 0.043 logMAR/year; *P* < 0.001), equivalent to two ETDRS letters. We observed a significantly faster decline of BVCA (*P* = 0.014) in patients with typical RP fundus appearance (0.060 logMAR/year; SE = 0.146) compared to patients with sine pigmento RP (0.011 logMAR/year; SE = 0.033). Moreover, BCVA declined significantly faster (*P* = 0.035) in patients with high myopia (0.081 logMAR/year; SE = 0.030) compared to those without high myopia (0.017 logMAR/year; SE = 0.039). Based on these observations, we then explored the combined effect of the sine pigmento RP and high myopia on BCVA. We found a significant slower BCVA decline (*P* < 0.05) in patients with sine pigmento RP and without high myopia (0.009 logMAR/year; SE = 0.004), followed by those with sine pigmento RP and high myopia (0.012 logMAR/year; SE = 0.0012). A faster BCVA decline was observed in patients that presented a typical RP fundus appearance in the absence of high myopia (0.020 logMAR/year; SE = 0.005), whereas the highest annual rate of BCVA change was observed in patients with a typical RP fundus and high myopia (0.098 logMAR/year; SE = 0.041). Finally, considering that patients with sine pigmento RP were significantly younger compared to patients with typical RP, we repeated the above analysis taking into account only observations within the same age range (i.e., 6 to 30 years) and we found a significantly (*P* < 0.001) faster decline of BCVA in patients with typical RP (0.024 logMAR/year; SE = 0.004) compared to patients with sine pigmento RP (0.007 logMAR/year; SE = 0.0006). Moreover, the regression model, including as covariate the age at the study baseline, confirmed a significantly faster progression (*P* = 0.017) in patients with typical RP compared to patients with sine pigmento RP.

**Table 2.** Main Findings of the Longitudinal Analysis of BCVA in Patients With *RPGR-*RP

	Mean Annual Rate			*P* Value
	(95% Confidence Interval)	Standard		(Comparison Between
Group	[logMAR/Year]	Error	*P* Value	Subgroups)
Overall cohort (best-seeing eye)^*^	0.025 (0.0004 to 0.050)	0.012	0.047	n.a.
Overall cohort	0.043 (0.023 to 0.063)	0.010	< 0.001	n.a.
Patients with typical RP	0.060 (0.031 to 0.088)	0.146	< 0.001	0.014
Patients with sine pigmento RP	0.011 (0.004 to 0.017)	0.033	0.001	
Patients with high myopia	0.081 (0.022 to 0.141)	0.030	0.007	0.035
Patients without high myopia	0.017 (0.009 to 0.025)	0.039	< 0.001	
Patients with sine pigmento RP and without high myopia	0.009 (0.008 to 0.010)	0.004	< 0.001	0.002
Patients with sine pigmento RP and with high myopia	0.012 (0.010 to 0.015)	0.0012	< 0.001	
Patients with typical RP and without high myopia	0.020 (0.010 to 0.030)	0.005	< 0.001	
Patients with typical RP and with high myopia	0.098 (0.017 to 0.179)	0.041	0.017	
Patients aged 6–30 years with typical RP	0.024 (0.016 to 0.032)	0.004	< 0.001	< 0.001
Patients aged 6–30 years with sine pigmento RP	0.007 (0.006 to 0.008)	0.0006	< 0.001	

* The progression is estimated on the worst-seeing eye unless otherwise specified.

n.a., non applicable.

**Figure 2.**
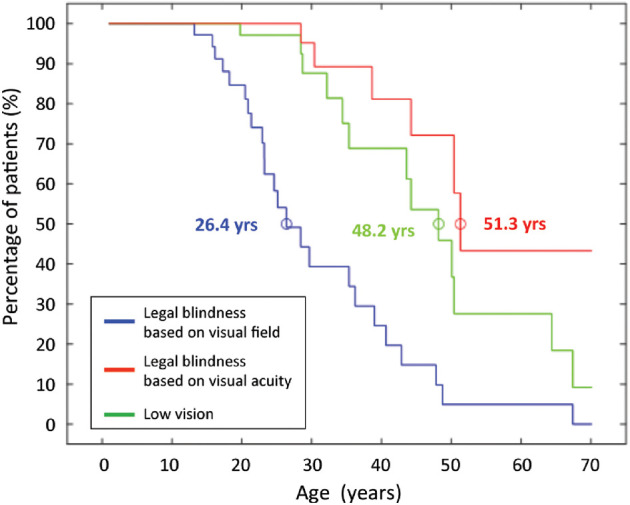
Survival curves of low vision and legal blindness (based on visual acuity and visual field) for patients with *RPGR* - associated RP. Kaplan–Meier plot showing the risk of developing low vision (*green*) and legal blindness, based on visual field (*blue*) or on visual acuity (*red*), with age. Survival analyses showed that legal blindness based on visual field is reached at a median age of 26.4 years (*blue circle*), low vision at 48.2 years (*green circle*), and legal blindness based on visual acuity at 51.3 years (*red circle*).

To assess the risk of developing low vision and blindness with ageing, a Kaplan-Meier curve analysis (hereafter referred to as “survival analysis”) was performed in the entire cohort. This analysis indicated that the patients reached low vision and blindness based on BCVA at the median age of 48.2 years and 51.3 years, respectively (Fig. 2). Survival analysis of blindness based on GVF suggested that the development of blindness was driven primarily by GVF loss. Specifically, the survival curve based on a GVF not greater than 10 degrees (median age = 26.4 years; 95% confidence interval [CI] = 21.1–31.6 years) was significantly shifted (*P* < 0.001) to younger ages when compared with a survival curve based on BCVA (median age = 51.3 years; 95% CI = 49.1–53.4 years).

Scotopic and photopic ERG responses were not detectable in 24 out of 48 patients. The remaining half showed a rod-cone pattern of photoreceptor dysfunction with undetectable scotopic and markedly abnormal photopic ERG responses. In particular, the mean b-wave amplitude of light-adapted 3.0 ERG was 20.7 ± 14.0 µV in right eyes and 24 ± 17.5 µV in left eyes, whereas the mean trough-to-peak amplitude of 30 Hz flicker ERG was 11.8 ± 9.1 µV in right eyes and 11.7 ± 10.7 µV in left eyes. Moreover, the light-adapted 3.0 ERG were delayed (normal range = 29–33 ms) with a mean implicit of 43.5 ± 2.8 ms in right eyes and 43.1 ± 4.4 ms in left eyes. Similarly, the 30 Hz flicker ERG showed longer implicit time than the normal range (46.6 ± 8.5 ms in right eyes and 45.8 ± 8.0 ms in left eyes; normal range = 25–31 ms).

The patients with a rod-cone pattern were significantly younger than those with undetectable ERG (24.8 ± 13.6 years versus 34.4 ± 15.5 years; *P* = 0.027). Moreover, a detectable photopic response was significantly associated with sine pigmento RP (13/18, 72.2% vs. 11/30, 36.7%; *P* = 0.018), also in the age-adjusted model (*P* = 0.032), whereas no association was found with high myopia (8/16, 50.0% vs. 16/32, 50.0%; *P* = 1.000).

Microperimetry examination, which was available for 24 patients (50.0%), showed a markedly reduced macular sensitivity (MS; 2.6 ± 3.3 dB in the right eyes; 3.8 ± 4.4 dB in the left eyes; normal value: 19.6 ± 0.4 dB). According to the evaluation in the eye with the best fixation stability, 17 patients (34.7%) showed stable fixation, 5 patients (10.2%) showed a relatively stable fixation, and 2 patients (4.1%) showed an unstable fixation. We did not identify any significant association of MS or fixation stability with age nor significant differences in their relationship with other clinical factors (i.e. high myopia and RP form).

Spectral domain OCT scans revealed MA in 27.1% of the patients analyzed (13/48). In particular, 12 patients (25.0%) had an ERM in at least one eye (7 bilateral; 5 monolateral) and one patient (2.1%) had a bilateral VMT. The patients with a MA were significantly older than patients without such alterations (39.5 ± 14.1 years vs. 25.9 ± 14.0 years; *P* = 0.005). The frequency of MA was similar in patients with and without high myopia (25.0% vs. 28.1%; *P* = 0.553) as well as in patients with typical RP and patients with sine pigmento RP (25.0% vs. 29.2%; *P* = 1.000). None of the patients presented a CME or had a history of CME (i.e. according to past medical records or in the OCT scans performed during the clinical follow-up).

Combined spectral domain OCT and fundus autofluorescence imaging were available for 28 patients (58.3%). The EZ band was detectable in all 28 patients and the mean width of the EZ band was 1319.0 ± 685.9 µm in the right eyes and 1326.0 ± 571.9 µm in the left eyes. The EZ band became significantly thinner with age at a linear rate of −27.5 µm/year (95% CI = −42.1 to −12.8 µm/year; *P* < 0.001) and at an exponential rate of −2.8%/year (95% CI = −4.4 to −1.1/year; *P* = 0.002). Spectral domain OCT scans acquired exclusively with the Heidelberg Spectralis OCTPlus with BluePeak showed that MMT, on average, was 226.7 ± 43.1 µm in the right eyes and 224.6 ± 44.4 µm in the left eyes. The progression of MMT with age did not reach statistical significance (*P* > 0.7) in both linear and exponential regression models. Analysis of follow-up data (available only in 8 patients) showed a loss of EZ band width at a linear rate of −183.7 µm/year (95% CI = −226.5 to −100.8 µm/year; *P* < 0.001) and at an exponential rate of −8.9%/year (95% CI = −12.8 to −4.8%/year; *P* < 0.001). Moreover, we observed a reduction of MMT at a linear rate of −5.8 µm/year (95% CI = −9.7 to −1.9 µm/year; *P* = 0.003) and at an exponential rate of −2.4%/year (95% CI = −4.2 to −0.6%/year; *P* = 0.011).

Based on the FAF imaging, we identified 14 patients (14/28; 50%) with a hyper-AF ring around the macula and no other significant FAF abnormalities. The remaining patients had a marked FAF decrease in the periphery with some degree of FAF in the posterior pole, whereas one patient showed an almost complete absence of FAF in the entire retina. We observed a significant difference when comparing the group of patients with a hyper-AF ring with those displaying a marked FAF decrease. The patients with a hyper-AF ring were younger than those with decreased FAF (17.5 ± 5.8 vs. 30.1 ± 14.6; *P* = 0.006; Table 3). They also had significantly better BCVA (β = −0.43 logMAR; *P* < 0.001) and a wider EZ band (β = 843 µm; *P* < 0.001). The frequency of MA was significantly lower in the patients with a hyper-AF ring compared to those with markedly decreased FAF. However, after correction for age, only the differences in EZ band width remained statistically significant (*P* < 0.001).

**Table 3.** Comparison Between Different FAF Patterns in Patients With *RPGR*-RP

Parameters	Hyper-AF Ring (*n* = 14)		Decreased FAF (*n* = 14)		*P* Value	
Age, y	17.5 ± 5.8		30.1 ± 14.6		0.006	
Self-reported age of onset, y	5.5 ± 3.6		7.4 ± 4.9		0.247	
Disease length, y	12.0 ± 5.8		22.7 ± 15.6		0.023	
Mean refractive error, D	−3.3 ± 0.7		−4.7 ± 1.0		0.277	
High myopia	4 (28.6%)		7 (50.0%)		0.220	
Typical RP	7 (50.0%)		7 (50.0%)		1.000	
Vitreomacular alteration	0 (0%)		5 (35.7)		0.020	
Detectable photopic ERG	10 (71.4%)		10 (71.4%)		0.590	
					Mean Difference	
					Estimation Beta (*P* Value)	
	Right Eye	Left Eye	Right Eye	Left Eye	Crude	Age-Adjusted
BCVA (logMAR)	0.15 ± 0.10	0.14 ± 0.09	0.41 ± 0.36	0.44 ± 0.34	−0.43 (< 0.001)	−0.08 (0.126)
EZ-band width, µm	1782.9 ± 556.9	1820.9 ± 702.6	855.1 ± 451.2	831.0 ± 390.9	843 (< 0.001)	823 (< 0.001)
MMT, µm	239.8 ± 39.6	237.4 ± 35.4	213.6 ± 43.8	211.9 ± 49.9	25.9 (0.089)	28.9 (0.076)
Photopic ERG (b-wave amplitude), µV	12.4 ± 17.3	15.3 ± 20.9	4.5 ± 8.1	4.3 ± 8.2	9.4 (0.067)	6.9 (0.138)
Photopic ERG (b-wave implicit time), ms	42.5 ± 3.1	42.6 ± 5.0	45.2 ± 1.7	44.7 ± 4.0	−2.4 (0.194)	−3.5 (0.096)
30 Hz Flicker ERG (trough-to-peak amplitude), µV	13.8 ± 14.5	12.6 ± 9.8	5.9 ± 4.1	7.0 ± 5.3	6.7 (0.066)	2.1 (0.455)
30 Hz Flicker ERG (implicit time), ms	44.2 ± 3.7	42.8 ± 2.4	50.9 ± 9.9	50.7 ± 9.8	−7.2 (0.011)	−8.4 (0.037)
MS, dB	4.8 ± 3.4	5.8 ± 3.6	2.2 ± 3.7	4.3 ± 5.6	2.0 (0.272)	2.4 (0.111)

### Molecular Characterization of the *RPGR*-RP Cohort

We identified *RPGR* causative variants in 48 male patients (from 31 unrelated families) diagnosed with RP. Almost one third of the patients were part of larger pedigrees that showed an X-linked inheritance, whereas the rest were simplex cases. The patients were analyzed either by panel-based sequencing of known retinopathy genes or by more comprehensive clinical exome sequencing. One patient (no. 41 in Table 4) was analyzed by Whole Exome Sequencing (WES). Following a first-tier next-generation sequencing (NGS) analysis, likely pathogenic variants in the *RPGR* exons 1 to 14 were identified in 23 patients at the hemizygous state (Table 4). The remaining 25 male patients included in this cohort were unsolved after NGS-based screening. Given that standard NGS-based approaches fail to accurately probe the *ORF15* mutational hot spot due to the presence of an extensive stretch of highly repetitive purine-rich sequence, we analyzed the latter subset of undiagnosed patients by direct PCR-based Sanger sequencing of the entire *ORF15* optimized for purine-rich sequences. We identified likely pathogenic variants in all cases. All the *RPGR* identified variants segregated with the RP phenotype in available family members.

**Table 4.** Variants Identified in the Patients With *RPGR*-RP

Family	Patient	RefSeq	Nucleotide Change	Protein Change	Location	Mutation Type	Reference
1	1	NM_001034853	c.139deI	p.(Ser47Leufs*21)	Exon 2	Frameshift	This study
19	35	NM_001034853	c.154G>A	p.?	Exon 2	Splice variant	^66^
25	42, 43	NM_001034853	c.154G>A	p.?	Exon 2	Splice variant	^66^
18	33, 34	NM_001034853	c.155-2A>G	p.(?)	Intron 2	Splice variant	^73^
14	29	NM_001034853	c.202G>C	p.(Gly68Arg)	Exon 3	Missense	^74^
30	48	NM_001034853	c.237_238del	p.(Val81Glnfs*6)	Exon 3	Frameshift	This study
15	30	NM_001034853	c.247+1G>A	p.(?)	Intron 3	Splice variant	This study
23	40	NM_001034853	c.439G>C	p.(Ala147Pro)	Exon 5	Missense	LOVD^†^
12	25, 26	NM_001034853	c.619+5G>A	p.(?)	Intron 6	Splice variant	^75^
5	10	NM_001034853	c.706C>T	p.Gln236*	Exon 7	Nonsense	^76^
17	32	NM_001034853	c.807T>C	p.(Cys250Arg)	Exon 7	Missense	^18^
20	36, 37	NM_000328	c.1059+1G>A	p.(?)	Intron 9	Splice variant	This study
2	2	NM_001034853	c.1106_1115del	p.(Arg369Glnfs*9)	Exon 10	Frameshift	This study
13	27	NM_001034853	c.1243_1244del	p.(Arg415Glyfs*37)	Exon 10	Frameshift	^77^
7	13	NM_001034853	c.1245+3A>T	p.(?)	Intron 10	Splice variant	^67^
24	41	NM_000328	c.1246-3A>G	p.(?)	Intron 10	Splice variant	This study
4	9	NM_001034853	c.1283dup	p.(Pro429Thrfs*24)	Exon 11	Frameshift	This study
29	47	NM_001034853	c.1345C>T	p.(Arg449*)	Exon 11	Nonsense	^78^
10	23	NM_001034853	c.1582_1585del	p.(Thr528Leufs*4)	Exon 14	Frameshift	^76^
16	31	NM_001034853	c.2232_2235del	p.(Asp744Glufs*70)	ORF15	Frameshift	This study
11	24	NM_001034853	c.2236_2237delGA	p.(Glu746Argfs*23)	ORF15	Frameshift	^23^
6	11, 12	NM_001034853	c.2311delG	p.(Glu771Argfs*44)	ORF15	Frameshift	This study
8	14–16	NM_001034853	c.2405_2406del	p.(Glu802Glyfs*32)	ORF15	Frameshift	^23^
26	44	NM_001034853	c.2405_2406del	p.(Glu802Glyfs*32)	ORF15	Frameshift	^23^
28	46	NM_001034853	c.2405_2406del	p.(Glu802Glyfs*32)	ORF15	Frameshift	^23^
21	38	NM_001034853	c.2466_2467del	p.(Lys823Argfs*11)	ORF15	Frameshift	^23^
22	39	NM_001034853	c.2706_2707del	p.(Glu903Glyfs*175)	ORF15	Frameshift	^79^
9	17–22	NM_001034853	c.2760_2761del	p.(Glu922Glyfs*156)	ORF15	Frameshift	^26^
27	45	NM_001034853	c.2792del	p.(Glu931Glyfs*158)	ORF15	Frameshift	^13^
31	49	NM_001034853	c.2993_2997del	p.(Glu998Glyfs*79)	ORF15	Frameshift	^23^
3	3–8	NM_001034853	ORF15 del	p.(?)	ORF15	Large deletion	This study

^†^Leiden Open Variation Database, variant entry # 0000575845.

**Figure 3.**
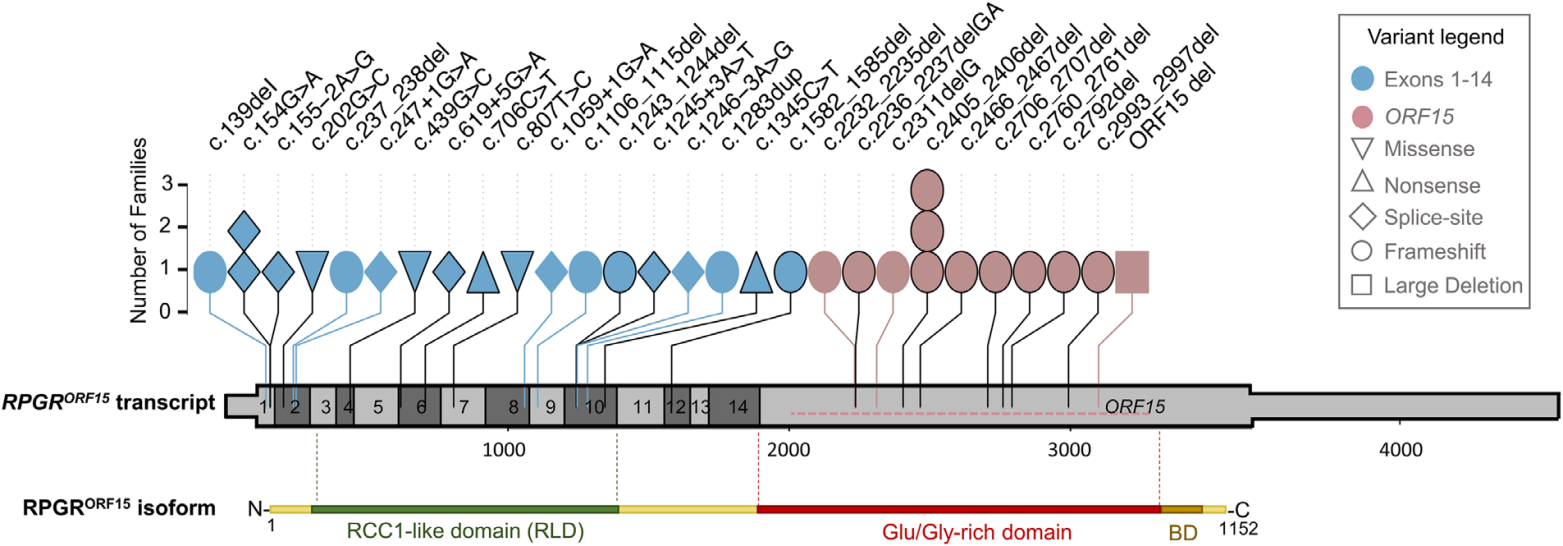
Genetic variants detected in *RPGR*. Schematic drawing of the *RPGR^ORF15^* transcript showing the position and frequency of the variants identified in this cohort. The untranslated regions of the transcript are depicted as a thinner bar. Each symbol designates a family whose variant position is reported on the *top*. The symbol color indicates whether the variant is found in exons 1 to 14 or in the *ORF15* region. The symbol shape indicates the mutation type. The large deletion (ORF15 del) is shown with a horizontal dotted line within the terminal exon of *RPGR^ORF15^.* Already reported variants are denoted with a *black border*. Protein domains of the retina-specific isoform RPGR^ORF15^ are shown below the transcript to indicate the RCC1-like domain (*green*), the Glu/Gly-rich region (*red*), and the basic domain (BD; *brown*; not to scale).

Half of our cohort harbored variants in *ORF15* (*n* = 25; 12 families; 52%), while the remaining part had causative variants in exons 1 to 14 (*n* = 23; 19 families; 48%; Fig. 3). In terms of type of mutations, frameshifts were the most frequently observed variants in this cohort (*n* = 25; 17 families; 52%), followed by splice variants (*n* = 12; 8 families; 25%), missense (*n* = 3; 3 families; 6%), and nonsense mutations (*n* = 2; 2 families; 4%; Fig. 3). We also identified an extended deletion of *ORF15* in 6 affected male subjects of a large pedigree (*n* = 6; 1 family; 13%). With the exception of this extended deletion, all variants detected in *ORF15* were deletions of few nucleotides (1 to 5 bases) causing frameshifts predicted to introduce premature termination codons. Ten of the 28 putatively pathogenic variants identified in this study were novel (Table 4) and had not been previously reported neither in the Human Gene Mutation Database (HGMD) nor in the Leiden Open Variation Database (LOVD). The frequency of these variants in reference population databases (e.g. ExAC, 1000 Genomes,^55^ and gnomAD) is compatible with a causative role in conditions with X-linked inheritance. The missense variant c.439G>C; p.(Ala147Pro) was previously reported as VUS in LOVD (variant entry #0000575845) and its potential pathogenicity was corroborated by in silico predictions (“Disease causing” in Mutation-taster; “Probably damaging” according to PolyPhen-2; category: deleterious, score: 0.01 according to Sorting Intolerant from Tolerant; score 26.6. in Combined Annotation-Dependent Depletion). This variant is absent from control population datasets, such as gnomAD (http://gnomad.broadinstitute.org) and Bravo (http://bravo.sph.umich.edu/).

### Genotype – Phenotype Correlation Analysis

To explore possible genotype-phenotype correlations, we stratified the unrelated patients according to the variant localization within the *RPGR^ORF15^* transcript (i.e. variants in exons 1–14 vs. variants in *ORF15*), as also implemented in other reports.^28^^,^^32^ We then assessed the disease severity of each patient group by considering the main clinical parameters of the cohort described herein (Table 5). We did not observe statistically significant differences in terms of patients’ age, self-reported age of onset, and disease length between patients with variants in exons 1 to 14 vs. those with variants in *ORF15* (Table 5). Similarly, there were no significant differences in the frequency of high myopia, MA, detectable photopic ERG, and the presence of a hyperautofluorescent ring. In contrast, patients harboring *ORF15* variants showed a faster loss of BCVA than patients with variants in exon 1 to 14 (*P* < 0.001). Specifically, in the former patient group, BCVA declined at a mean annual rate of 0.044 logMAR/year (equivalent to 2 ETDRS letters/year), whereas the mean rate of decline in the latter group was 0.011 logMAR/year (equivalent to 1/2 ETDRS letters/year). On average, patients with *ORF15* variants showed lower values of EZ band width, MMT and MS compared to those with variants in exons 1 to 14, but these differences were not statistically significant (Table 5). None of the patients showed significant extra-ocular findings with the exception of five unrelated patients (nos. 2, 9, 32, 37, and 40) carrying mutations in the exons 1 to 14, who reported recurrent otitis during childhood.

**Table 5.** Clinical Features of Patients With *RPGR*-RP Stratified According to the Localization of the Variant (Exons 1–14 vs. *ORF15*)

	Exon 1–14		*ORF15* (12 Unrelated			
Parameters	(19 Unrelated Patients)		Patients)		*P* Value	
Age, y	26.2 ± 12.5		33.3 ± 19.1		0.223	
Self-reported age of onset, y	6.3 ± 5.0		7.6 ± 5.4		0.492	
Disease length, y	20 ± 12.6		25.7 ± 18.9		0.321	
Mean refractive error, D	−3.9 ± 2.9		−3.6 ± 3.4		0.774	
High myopia	7 (36.8%)		3 (25.0%)		0.697	
Typical RP	10 (52.6%)		9 (75.0%)		0.274	
Vitreomacular alteration	4 (21.1%)		2 (16.7%)		1.000	
Detectable photopic ERG	10 (52.6%)		6 (50.0%)		1.000	
Hyperautofluorescent ring	6 of 12 (50.0%)		3 of 7 (42.9%)		1.000	
Annual BCVA decline (best-seeing eye)	0.011 ± 0.004 (*P* = 0.003)		0.044 ± 0.001 (*P* < 0.001)		< 0.001	
					Mean Difference	
					Estimation Beta (*P* Value)	
	Right Eye	Left Eye	Right Eye	Left Eye	Crude	Age-Adjusted
BCVA, logMAR	0.34 ± 0.42	0.34 ± 0.41	0.76 ± 0.79	0.86 ± 0.81	0.47 (0.044)	0.32 (0.063)
EZ-band width, µm	1492 ± 634	1518 ± 700	1184 ± 715	1224 ± 796	−301.1 (0.345)	−216.2 (0.469)
MMT, µm	239.6 ± 45.8	240.0 ± 44.1	205.7 ± 41.0	205 ± 44.7	−34.4 (0.075)	−34.1 (0.095)
Photopic ERG (b-wave amplitude), µV	17.8 ± 8.1	23.9 ± 16.4	28.3 ± 32.6	30.8 ± 33.7	8.68 (0.613)	−5.65 (0.706)
Photopic ERG (b-wave implicit time), ms	43.5 ± 1.9	43.5 ± 3.0	44.0 ± 1.1	41.6 ± 2.5	1.6 (0.442)	1.3 (0.571)
30 Hz Flicker ERG (trough-to-peak amplitude), µV	10.2 ± 8.5	10.1 ± 6.3	14.3 ± 17.4	15.7 ± 15.1	4.8 (0.451)	4.42 (0.305)
30 Hz Flicker ERG (implicit time), ms	48.6 ± 10.5	48.7 ± 9.9	49.9 ± 5.5	37.2 ± 7.7	−3.5 (0.245)	−3.1 (0.299)
MS, dB	2.9 ± 3.5	4.3 ± 4.8	2.5 ± 4.1	2.4 ± 5.3	−1.18 (0.58)	−0.87 (0.696)

We then sought to further classify the variants in exons 1 to 14 into three groups according to the mutation types and their predicted consequence at the protein level (i.e. missense variants [M], splice-site variants [S], and null variants [N] that comprised nonsense and frameshift changes; Supplementary Table S2). Using this stratification, we found a differential effect of variants in exons 1 to 14. Patients bearing missense variants (*n* = 3) presented the mildest phenotypes, considering also their older mean age, the relatively preserved mean visual acuity, and the highest mean values of EZ band and MS (Supplementary Table S2). Moreover, the age-adjusted model revealed a reduction of flicker ERG responses in patients carrying null variants compared to the *ORF15* group (*P* < 0.001), in spite of a better-preserved macular thickness (i.e. higher MMT values). The slower BCVA decline detected in patients harboring splice-site variants compared to patients with null variants (i.e. 0.008 logMAR/year vs. 0.011 logMAR/year) was not statistically significant (*P* = 0.468).

Overall, our data indicated that mutations in *ORF15* are associated with a more severe phenotype compared to variants in exons 1 to 14.

## Discussion

This study describes the natural history of RP due to *RPGR* mutations in a large Italian cohort (48 male patients from 31 different families) evaluated at a single center with a long-term follow-up (mean = 6.5 years). To our knowledge, this is the first report of a longitudinal natural history study in Italian patients with *RPGR*-associated RP that includes detailed morphological and functional assessments. The largest cohort of RP patients with *RPGR* mutations (113 patients) was characterized by Sandberg et al.^29^ focusing mainly on visual acuity, visual field, and ERG assessments in US patients. More recently, Talib et al. described a multicenter, retrospective cohort study involving 52 patients with *RPGR*-RP from various Dutch medical centers.^32^ However, as the authors also pointed out, the potential intercenter variability (which may not always be statistically accounted for in the retrospective study setting) may have complicated data interpretation.^32^ With respect to previous reports, our results extend the number of patients followed up at a single referral center and provide a comprehensive and homogeneous clinical description of *RPGR*-associated forms of RP. In this RP cohort, symptoms were first reported at 5 years of age, thus confirming the earlier onset of *RPGR*-RP, particularly compared to autosomal dominant RP forms.^56^^,^^57^ About 80% of patients presented variable degrees of myopia, and approximately one third of the cohort had high myopia, in line with previous observations in X-linked RP^56^^,^^57^ and in *RPGR*-related RP.^32^ Our data confirm a faster BCVA decline in patients with high myopia as opposed to those without high myopia.^32^ Moreover, the patients with typical RP and high myopia had a significantly faster progression of the disease compared to those with sine pigmento RP in the absence of high myopia. In terms of photoreceptor function, the sine pigmento RP forms were significantly associated with detectable photopic ERG responses. These findings suggest that the scarcity or absence of pigment deposits could be considered as a prognostic factor for a slower disease progression, particularly in the absence of high myopia and in the first three decades of life.

The survival analyses over an extended follow-up period showed that the BCVA decline led to blindness at a median age of 56 years. Blindness was mainly driven by visual field loss. In particular, patients reached blindness based on visual field at a significantly younger age (26.4 years; i.e. about 25 years earlier), than blindness based on BCVA. These findings are consistent with the report of Sandeberg et al.^29^ showing that blindness based on visual field was achieved about 10 years earlier than blindness based on BCVA. It should be noted though that the different thresholds in the definition of blindness by BCVA could impact the above comparison: specifically, Sandberg et al.^29^ adopted the value of 20/200, whereas we considered a visual acuity of 20/400 (according to the ICD-10 version 2016).

FAF imaging revealed the presence of a hyperautofluorescent ring in 14 patients (50%; 14/28) that were significantly younger and had a better preserved EZ band compared to patients displaying other FAF abnormalities (i.e. marked reduced macular FAF or absence of macular FAF). On this basis, it is tempting to hypothesize that FAF patterns could be indicative of different stages of disease progression. Specifically, the hyperautofluorecent ring may represent the front of advancing concentric photoreceptor cell loss, as previously shown in RP in general^45^ and in subjects with *ORF15* variants.^34^

Finally, in terms of MA, we did not detect CME in any of our patients with *RPGR*-RP even though CME is the most frequently encountered MA in our general RP cohort, affecting 22.9% of patients.^58^ This finding is consistent with two previous studies that reported no case of CME in patients with *RPGR*-RP.^9^^,^^35^ Moreover, the lower frequency of CME observed in patients with X-linked RP (7.1%) compared to autosomal forms reported in our previous study^58^ strongly suggest that the incidence of CME in RP varies according to the mutated gene. On the contrary, the frequency of ERM and VMT in patients with *RPGR-*RP was similar to that observed in our overall RP cohort (ERM = 25% in *RPGR*-RP vs. 19.8% in RP; VMT = 2.5% in *RPGR*-RP vs. 5% in RP).^58^ The prevalence of ERM in our cohort was lower compared to that reported by Talib et al. (i.e. 40% in at least one eye).^32^ A possible explanation for this difference could be due to a selection bias, since only 29% (15/52) of their cohort underwent spectral domain OCT (compared to 100% in this study, 48/48). Moreover, even if longitudinal observations of spectral domain OCT scans acquired with the Heidelberg Spectralis and follow-up mode were available only for small subgroups of patients, the analysis showed a significant loss of the EZ band width at an annual rate of −183.7 µm/year and at an exponential rate of −8.9%/year. These estimates are comparable with the values reported in previous studies^44^^,^^59^ on X-linked patients, which range from −210 µm/year to −270 µm/year and from −7% to −9.6%/year. This evidence supports the adoption of the EZ band width as a relevant outcome measure for the evaluation of disease progression and of treatment efficacy in patients with *RPGR*-RP.

We identified 10 novel *RPGR* variants, expanding the list of *RPGR* disease-causing mutations (see Table 4). Among those, two novel single nucleotide variations (SNVs) and a large deletion were found within the *ORF15.* All *ORF15* variants were identified by direct Sanger sequencing of this region in patients that remained undiagnosed after a panel-based NGS analysis, suggesting that alternative ad hoc methods (either Sanger sequencing^34^^,^^50^^,^^60^ or NGS-based^61^^,^^62^) should be deployed for accurate probing of *ORF15* in unsolved cases after a first-tier NGS analysis (especially in men with XL or simplex RP/CD/CRD).

Several attempts have already been made to correlate the genotype with disease severity in patients carrying *RPGR* mutations, but conclusions are not always concordant. For example, a more severe clinical phenotype has been associated with variants in exon 1 to 14 in some cohorts.^28^^,^^30^^,^^63^^,^^64^ In contrast, other studies argue that variants in the *ORF15* region of the *RPGR* gene present with a more deleterious phenotype.^31^^,^^32^ Cohort's size, selection bias, variability of patients' diagnoses (e.g. inclusion of both patients with RP and patients with CRD) as well as the potential nonuniformity of observations made in multicenter studies, represent some factors that may impact on the phenotypic-genotypic correlations inferred. In this study, we classified the variants according to their position within the *RPGR^ORF15^*. We therefore explored possible correlations between the clinical parameters of patients with RP and the variant location in *RPGR* in this cohort. We found that variants in *ORF15* correlated with a more severe phenotype in terms of BCVA loss. In particular, patients carrying *ORF15* variants had a faster decline of BCVA compared with patients with mutations in exons 1 to 14. Moreover, these patients showed lower values of MMT compared with patients harboring variants in exons 1 to 14, albeit this was not statistically significant. These findings partially corroborate the recent observation that variants in *ORF15* correlated with a more severe visual impairment, including higher myopia, a thinner central retina, and faster progression of visual field loss.^32^ The disease severity associated with *ORF15* variants could be attributed to the presumed gain-of-function or dominant-negative effect of the resulting protein product, as previously suggested.^28^^,^^30^^,^^35^^,^^40^ Variants in *ORF15* are mostly small deletions or duplications leading to frameshift changes. Because *ORF15* is the terminal exon of the *RPGR^ORF15^* transcript, the stop or frame-shifting variants are, in principle, not subject to nonsense-mediated mRNA decay, but can be expected to lead to the synthesis of presumably dysfunctional protein products. Such proteins could severely perturb RPGR protein networks and exert a more deleterious effect compared to that of null variants in exons 1 to 14. Indeed, in vivo studies using transgenic mice demonstrated that certain truncated forms of *RPGR* behave as gain-of-function mutants and can be more deleterious than null alleles.^65^

By further stratifying patients with variants in exons 1 to 14 based on the mutation type, we noted that splice-site variants in this region were associated with milder phenotypes compared to null alleles (Supplementary Table S2). Given that the consequences of splice-site variations can vary greatly, we cannot exclude that this observation may be biased by the small number of splice variants in our cohort. In addition, two of the splice-site variants (c.154G>A and c.1245+3A>T) had already been shown to cause in-frame changes, namely, an in-frame deletion of 42 amino acids^66^ and in-frame skipping of exon 10.^67^ Therefore, to reliably interpret genotype-phenotype associations involving *RPGR* splice-altering variants, in vitro analyses of their consequence at the protein level should be pursued, as also performed for other genes (e.g. *ABCA4*).^68^ In this context, it is interesting that in patient-derived cell lines the consequence of the c.1245+3A>T splice site variant (exon 10 in-frame skipping) was shown to result in a strongly reduced localization of the RPGR protein along the cilium,^39^ suggesting a possible read-out assay to quantify the severity of *RPGR* mutations or efficacy of treatment approaches.

Considering the above points, further studies are required to establish clear correlations between *RPGR* variants and their impact on visual function and retinal morphology, especially given the phenotypic variability of *RPGR* patients. Some reports describe extensive phenotypic variability in families harboring the same variants in *RPGR*, as well as different diagnosis (i.e. XLRP and XLCRD)^8^^,^^28^^,^^69^^,^^70^ even in dizygotic twins within the same kindred.^71^ Clearly, much remains to be understood about the contribution of genetic modifiers, environmental effects, and their synergistic effects on the observed phenotypic variation. Therefore, any attempt to predict the clinical severity or disease progression on the basis of the *RPGR* mutation type or location should cautiously consider the complex interplay of different factors.

The current study has some limitations mainly related to its retrospective design. First, although the patients were invited to undergo yearly follow-up visits, only cross-sectional data are available for some patients and for some outcome measurements. Moreover, MP1 measurements are available for a subgroup of patients, because MP1 was introduced in our clinical practice only recently and some patients were not always willing or capable to perform the examination (e.g. because of poor visual acuity or young age). For similar reasons, also fundus autofluorescence was available only for a subset of patients. Finally, the follow-up length is not the same for all the patients and the relatively small sample size may hinder the detection of statistically significant associations in some comparisons due to the insufficient power of the test.

In conclusion, we present a single-center, retrospective, longitudinal study that comprehensively describes visual function and disease progression in subjects with *RPGR*-associated RP on the grounds of morphological and functional parameters. Based on the longitudinal analysis, we identified a subgroup of patients with *RPGR*-associated RP who showed a more severe phenotype and faster disease progression. These patients were older, had typical RP, elevated myopia, and harbored pathogenic variants in the *ORF15* sequence. On the other hand, we defined a subgroup of patients presenting an overall milder phenotype with a better visual acuity, a better-preserved EZ band, the presence of an AF ring, fewer MA, and a slower disease progression. These were patients of younger age, with sine pigmento forms of RP, no myopia, and variants in exons 1 to 14. Taken together, these results suggest that the selection and prioritization of patients for clinical trials should consider the patient's age, the RP form according to the fundus appearance, the presence of high myopia, as well as the localization and type of causative variants in the *RPGR* gene. Finally, this study adds new knowledge on the disease and provides a novel resource to guide patient selection and the design of outcome measures in recently opened RPGR treatment trials.^72^

## Supplementary Material

Supplement 1
