# Supplementary material for: Spectrum of Disease Severity in Patients With X-Linked Retinitis Pigmentosa Due to RPGR Mutations
Source: Invest Ophthalmol Vis Sci. 2020 Dec 29;61(14):36. doi: 10.1167/iovs.61.14.36 (PMC7774109; doi:10.1167/iovs.61.14.36)
Supplement: Supplement 1 [file iovs-61-14-36_s001.pdf]

SUPPLEMENTARY MATERIAL

Supplementary Table 1. List of primer sequences used for the *RPGR*-ORF15 amplification and Sanger sequencing

Primer	Sequence (5'- 3')	T_a (°C)	T_e (°C)
R1Ampl	GACTAAACCCATAATATCCAAATCCA	60	72
R2Ampl	GCCAAAATTTACCAGTGCCTCCTAT	60	72
R4Seq	CTCTCCTTCCTCCTTTTCAC	56	60
R5Seq	ACTGGCCATAATCGGGTCACAT	56	60
R7bSeq	CCTTCCTCCTCTTCCCCCTCA	58	63
R8bSeq	TCCTTCCTCCTCTTCCCCCTCCCA	63	68
R9Seq	CCCTGTGTGTTAGTAACTGAC	56	60
R11Seq	CAATTTAATAACACGTAATGAGTG	56	60

T_a, annealing temperature; T_e, extension temperature

Supplementary Table 2. Clinical features of the patients with variants in exon 1-14 stratified according to the mutation type

Parameters	Missense (M; 3 unrelated patients)		Splice-site (S; 8 unrelated patients)		Null (N; 8 unrelated patients)		*p**	
Age (years)	36.1 ± 27.1		21.5 ± 7.2		27.3 ± 8.3		0.197	
Self-reported age of onset (years)	5 ± 1		6.1 ± 4.3		6.9 ± 6.7		0.845	
Disease length (years)	31.1 ± 26.3		15.4 ± 5.9		20.4 ± 10.1		0.265	
Mean refractive error (D)	-5.5 ± 2.3		-3.3 ± 3.4		-3.9 ± 2.5		0.930	
High myopia	2 (66.7%)		3 (37.5%)		2 (25%)		0.804	
Typical RP	1 (33.3%)		5 (62.5%)		4 (50%)		0.516	
Vitreomacular alteration	1 (33.3%)		1 (12.5%)		2 (25%)		0.800	
Detectable photopic ERG	1 (33.3%)		5 (62.5%)		4 (50%)		0.836	
Hyperautofluorescent ring	2 out of 2 (100%)		2 out of 6 (33.3%)		2 out of 4 (50%)		0.865	
Annual BCVA decline (best-seeing eye)	not computable		0.008 ± 0.001 ($p < 0.001$)		0.012 ± 0.005 ($p = 0.018$)		<0.001 (for comparisons vs *ORF15*) 0.468 (for S vs N)	
	Right eye	Left eye	Right eye	Left eye	Right eye	Left eye	*p* (Age-adjusted models): S vs *ORF15*	N vs *ORF15*
BCVA (logMAR)	0.44 ± 0.49	0.47 ± 0.47	0.21 ± 0.12	0.2 ± 0.10	0.43 ± 0.59	0.44 ± 0.55	0.005 (0.015)	0.261 (0.313)
EZ-band width (μm)	1985 ± 208	2045 ± 285	1337 ± 322	1385 ± 335	1478 ± 233	1453 ± 325	0.691 (0.436)	0.455 (0.160)
MMT (μm)	248.0 ± 32.5	247.0 ± 26.9	216.8 ± 21.5	214.3 ± 17.6	269.5 ± 21.0	275.0 ± 28.7	0.692 (0.677)	<0.001 (0.001)
Photopic ERG (b-wave amplitude) (μV)	10.2	26.6	22.5 ± 8.9	28.8 ± 23.5	14.5 ± 4.5	15.3 ± 1.8	0.165 (0.053)	0.380 (0.097)
Photopic ERG (b-wave implicit time) (ms)	44.9	45.2	43.5 ± 2.2	44.2 ± 3.0	41.6 ± 3.9	42.9 ± 2.5	0.381 (0.570)	0.413 (0.478)
30 Hz Flicker ERG (trough-to-peak amplitude) (μV)	11.5	15.8	12.1 ± 11.1	11.8 ± 7.3	7.1 ± 4.1	6.0 ± 2.1	0.666 (0.367)	0.173 (<0.001)
30 Hz Flicker ERG (implicit time) (ms)	42.6	47.3	52.6 ± 13.5	52.0 ± 13.9	45.1 ± 5.8	45.1 ± 1.9	0.800 (0.891)	0.219 (0.257)
MS (dB)	4.1 ± 3.7	5.2 ± 4.0	3.3 ± 4.2	5.0 ± 6.3	2.0 ± 3.0	3.1 ± 3.6	0.517 (0.849)	0.954 (0.943)

*: comparison between groups S, N and *ORF15*
